# miR-125b Enhances IL-8 Production in Early-Onset Severe Preeclampsia by Targeting Sphingosine-1-Phosphate Lyase 1

**DOI:** 10.1371/journal.pone.0166940

**Published:** 2016-12-09

**Authors:** Weiwei Yang, Anning Wang, Chunling Zhao, Qinghua Li, Zhifang Pan, Xuefu Han, Cuijuan Zhang, Guohui Wang, Chao Ji, Guili Wang, Guangtao Jia, Jiyu Ju, Wei Gao, Wenjing Yu, Xiaoying Liu, Xi Chen, Weiguo Feng, Zhiqin Gao, Jie Li, Chune Ren

**Affiliations:** 1 School of Biological Sciences, Weifang Medical University, Weifang, China; 2 Department of Cardiology, Weifang People’s Hospital, Weifang, China; 3 Public Health College, Weifang Medical University, Weifang, China; 4 Department of Cardiology, Weifang People’s Hospital, Weifang, China; 5 Department of Obstetrics, Affiliated Hospital of Weifang Medical University, Weifang, China; 6 Center for Reproductive Medicine, Affiliated Hospital of Weifang Medical University, Weifang, China; 7 Department of Dentistry, Weifang Maternity and Child Care Hospital, Weifang, China; 8 Biopharmaceutical Laboratory of Health and Family Planning Commission of Shandong Province, Weifang Medical University, Weifang, China; University of Texas MD Anderson Cancer Center, UNITED STATES

## Abstract

Preeclampsia (PE) is one of the leading causes of maternal and perinatal mortality and morbidity. One of the main hallmarks observed in PE is impaired inflammation state. In the current study, we found that miR-125b was deregulated in placental tissues and plasma derived from PE patients, which suggest a potential association between this miRNA and the pathogenesis of PE. Overexpression of miR-125b significantly reduced SGPL1 expression, and luciferase assays confirmed that SGPL1 is a direct target of miR-125b. We also found that miR-125b enhanced IL-8 production by directly targeting sphingosine-1-phosphate lyase 1 (SGPL1), and this effect could be reversed by SGPL1 overexpression. In placentas derived from PE patients, a negative correlation of miR-125b and SGPL1 was observed, and IL-8 was validated to be increased in the circulation of PE patients. Our data demonstrated a critical role of miR-125b in IL-8 production and the development of PE.

## Introduction

Preeclampsia (PE) is the leading cause of adverse health problems and morbidity in both the mother and the fetus worldwide[1], which is characterized by new occurrence of high blood pressure and proteinuria after the 20^th^ week of pregnancies. Despite intensely investigation, the etiology of PE remains unclear[1,2]. However, it is widely accepted that the placenta plays a central role in the progress of PE, as delivery of the placenta leads to elimination of the phenomenon[2]. Altered release of placenta soluble factors is hypothesized to induce maternal systematic dysfunction, resulting in the clinical findings of hypertension and proteinuria in PE patients[3,4].

PE has been grouped into early-onset (before 34+0 weeks) and late-onset (after 34+0 weeks) PE recently. Although the presenting features overlap, it was widely accepted that early-onset and late-onset PE have different etiologies and therefore different clinical complications[5]. Comprising a small subset of all PE cases (5% ~ 20%), early-onset PE represents the most severe cases of respective clinical relevance, and has long been the focus of basic and clinical research. Early-onset PE is characterized by an increased systemic inflammation caused by a deregulated immune balance[6].

MicroRNAs (miRNAs) are endogenous short regulatory RNAs, usually 22~24 nucleotides long, which directly regulate gene translation by pairing with the 3’-untranslated region (UTR) of target transcripts, resulting in translational suppression or degration of target mRNA transcript[7]. Critical roles of miRNAs have been investigated for many aspects of development, homeostasis and disease[8]. Although previously studies reported over 600 miRNAs expressed in human placenta and indicated distinct genome-wide expression profile of miRNAs between normal and PE placentas[9–13], their roles in the human placenta and the pathogenesis of PE are not fully cleared. Research on deregulated miRNAs in the pathogenesis of PE could contribute to interpret the underlying mechanism of the syndrome and develop novel predicting biomarkers and intervention targets for the disease.

*Hsa-miR-125b-5p* (miR-125b) is member of miR-125 miRNAs family that involves in the immune response, angiogenesis or oxidative stress[14–16]. miR-125b has been found to play essential roles in the development of kinds of cancers[14,15,17,18]. Our microarray data suggest that miR-125b may be associated with severe PE[19]. In situ hybridization studies also revealed that miR-125b was localized in trophoblasts in the placenta[20,21]. However, functions of miR-125b in human placenta and PE remain poorly understood.

In the current study, we aimed to explore the possible association of miR-125b and the pathology of PE, especially early-onset PE. For that purpose, we characterized the level of miR-125b in the circulation and placenta tissues of PE patients, and identified the direct target gene of miR-125b in PE placenta. We further investigated the roles of miR-125b in the placenta and the pathology of the disorder.

## Materials and Methods

### Patients and samples collection

Placenta samples were collected from pregnant women who give birth in the Department of Obstetrics of Affiliated Hospital of Weifang Medical University and Weifang Maternity and Child Care Hospital. PE was diagnosed by the definition in Williams Obstetrics (23^rd^ edition) as previously described[19]. The patients enrolled in our study were diagnosed as early-onset severe PE. Briefly, patients had *de-novo* occurrence of systolic blood pressure (SBP) ≥160 mm Hg or diastolic blood pressure (DBP) ≥110 mm Hg on at least two occasions, accompanying severe proteinuria (≥3+ or ≥2 g/24h) at 20–34 weeks of gestation. The symptoms including hypertension and proteinuria of all patients were eliminated 6 weeks postpartum. For the normal pregnant group, women with any other complications during pregnancy were excluded from this study. Placenta tissues at the chorionic and basal plate were obtained separately from the central part of placenta within 1 hour of Cesarean birth. The samples were washed in saline to remove blood and stored in liquid nitrogen immediately before bench work.

We collected samples from 57 pregnancies, 17 of which were PE patients. As presented in Table 1, there are no significant variations in age, body mass index (BMI), glucose tolerance (indicated by 50g GCT) and nulliparous percentage between normal pregnancy women and women with PE enrolled in our current study. However, the systolic blood pressure (SBP), the diastolic blood pressure (DBP) and the proteinuria of PE patients are significantly higher than those of the normal controls.

**Table 1:** Clinical characteristics of patients enrolled in the research. We found no significant variations in age, BMI, GCT, infant birth weight and nulliparous percentage between the preeclampsia patients and the normal control pregnant women enrolled in our current study. However, blood pressure (SBP and DBP) and proteinuria (24-hour urine protein) of preeclampsia patients is significantly higher than those of controls. The preeclampsia patients delivered earlier and had relative low weight babies. Data are shown as mean ± SEM, and differences between normal and SP patients were analyzed with Student-t test. BMI, body mass index; 50g GCT, 50g glucose tolerance test; SBP, systolic blood pressure; DBP, diastolic blood pressure; NA, not available.

	Normal (N = 40)	SPE (N = 17)	*p*-value
Age, y	28.96 ± 4.11	28.85 ± 2.02	0.9170
BMI, kg/m^2^	22.69 ± 2.60	22.83 ± 2.18	0.8462
SBP, mm Hg	111.9 ± 11.38	162.3 ± 7.8	< 0.0001
DBP, mm Hg	76.57 ± 5.70	104.2 ± 4.5	< 0.0001
Proteinuria, g/24h	NA	4.40 ± 1.36	NA
50g GCT, mM	6.51 ± 1.45	6.63 ± 1.48	0.2183
Nulliparous, %	81.3	83.9	NA

All works were carried out in accordance with the approved guidelines. All participants provided their written informed consents, and the consent forms and all experimental protocols were approved by the Ethics Committee of Weifang Medical University, reference number 2014/041.

### Cell culture

The human trophoblast cell line, HTR8/SVneo cells, was obtained from Dr. Charles H. Graham at Queen’s University, Canada[22]. Cells were cultured under 5% CO_2_ at 37°C according the supplier’s protocols using RPMI1640 medium (Invitrogen, CA, USA) supplemented with 10% fetal bovine serum (FBS).

### RNA extraction and Real-time qPCR

Total RNA was extracted using TRIzol^®^ reagent (Invitrogen, CA, USA) according to the manufacture’s protocol and tested on a NanoDrop Spectrophotometer (Thermo Scientific). Reverse transcription were performed using PrimeScriptTM RT reagent Kit (Takara, Dalian, China) or miRcute miRNA First-strand cDNA Synthesis Kit (Tiangen, Beijing, China).

To examinie miR-125b expression, Real-time qPCR was carried out using a miRcute miRNA qPCR Detection kit (Tiangen, Beijing, China) and normalized to either the small nuclear RNA U6 (snU6) for cells and tissures. For plasma, the synthetic *Caenorhabditis elegans* miR-39 (Cel-miR-39) (Qiagen) was added in the isolation of total RNA from plasma and served as normalization control as describled by Kroh et al[23]. PCR programs were set up as follows: initial denaturation 5 min at 95°C and followed by 40 cycles with denaturation 20s at 95°C and annealing 34s at 60°C according to the manufacture’s manual.

To determine relative expression of SGPL1, Real-time qPCR was performed using the SYBR Premix Ex Taq kit (Applied Biosystems, CA, USA) according to the manufacturer’s manuals with GAPDH using as internal control. Real-time qPCR reaction was conducted using the ABI PRISM 7500 sequence detection system (Applied Biosystems, Carlsbad, CA, USA), the thermocycling conditions were as follows: 5 min at 95°C followed by 40 cycles with 5s at 95°C and 31s at 60°C. Detection of each sample were replicated at least 3 times.

### Western blotting

Proteins were prepared using Radioimmunoprecipitation assay buffer (RIPA) as previously reported[24]. Lysate protein concentrations were measured by BCA Assay (Pierce, Rockford, IL, USA). For Western blotting, protein extracts were separated by 10% SDS-PAGE and transferred to the nitrocellulose membranes. The membranes were incubated with primary antibodies overnight at 4°C after blocking. The primary antibodies used were mouse anti-human SGPL1 (Santa Cruz, Santa Cruz, CA, USA), goat anti-human IL-8 (Abcam, CA, USA) and mouse anti-human GAPDH (Santa Cruz Biotechnology, Santa Cruz, CA, USA); and anti-mouse-HRP-conjugated secondary antibody was used as secondary antibody (Invitrogen, CA, USA). The signals were detected using an Enhanced Chemiluminescence Plus kit (Amersham, NJ, USA) and visualized after exposure to a Kodak film. Relative densities of SGPL1 and IL-8 were normalized to GAPDH of the same blot. The band intensity were analyzed by Image J v1.50 (NIH, USA).

### Immunohistochemistry

Placenta samples were fixed in 4% paraformaldehyde (Sigma, MO, USA) and embedded in paraffin wax. The tissue was then cut into to 5μm sections. The sections were subjected to deparaffinization, rehydration and antigen recovery before being incubated with antibodies against human SGPL1 (Santa Cruz Biotechnology, Santa Cruz, CA, USA) overnight at 4°C. The sections were then incubated with HRP-conjugated secondary antibody (Zhongshan Goldenbridge Biotechnology Co., Beijing, China) for 30 min at room temperature and visualized with DAB.

### Enzyme Linked-Immuno-Sorbent Assay (ELISA)

Concentrations of IL-8 in plasma and cell supernatant were examined by using Enzyme Linked-Immuno-Sorbent Assay (ELISA) according to the manufacturer’s manual (R&D, CA, USA). Briefly, 50 μl of each sample were used for assay, and all samples were assayed in duplicate. The concentrations of the samples were determined according to the absorbance at 450-nm wavelength in a microplate reader, while wavelength correction was set to 570 nm.

### Luciferase assays

The sequence in the 3’-UTR segments of human SGPL1 gene and a sequence with mutations of two nucleotides in the miR-125b target site were cloned into pMiR-Report vector to produce the recombinant vectors, pmir-SGPL1 and pmir-SGPL1-M, respectively. The vectors containing the wide type or mutant 3’-UTR of SGPL1 and miR-125b mimics were co-transfected into HTR8/SVneo cells. Luciferase activity was determined using the Dual-luciferase Reporter Assay System according to the manufacturer’s manuals (Promega, WI, USA) 48h post co-transfection.

### Statistical analysis

All experiments were triplicate repeated independently in identical conditions. Results are shown as means ± SEM. Statistical analysis was performed using SPSS statistics software (IBM, NY, USA), with *p* < 0.05 considered as significant.

## Results

### miR-125b is elevated in PE patients

**Fig 1:**
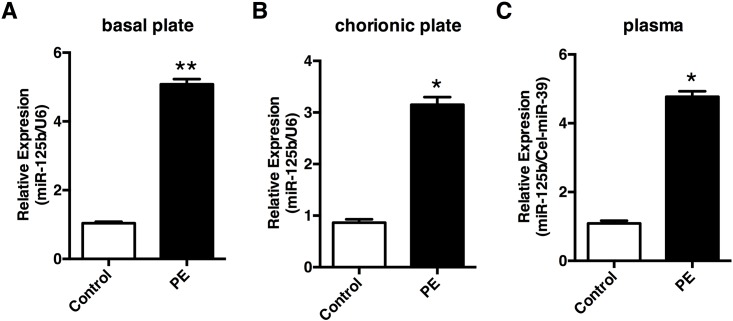
Expression of miR-125b in the plasma and placentas from preeclampsia patients (PE) and normal control pregnancies (NC). Relative expression of miR-125b was measured in the basal plate **(A)** and chorionic plate **(B)** of placentas from preeclampsia patients and normal controls by Real-time qPCR, with the small nuclear RNA U6 as internal control. Circulating levels of miR-125b was tested in plasma from preeclampsia patients and normal controls, taking the synthetic Caenorhabditis elegans miR-39 (Cel-miR-39) as external control. All experiments were repeated ≥3 times independently in identical conditions. Results are presented as mean ± SEM. Statistical comparison between PE group and NC group was performed using SPSS, with p<0.05 considered as significant. *p < 0.05.

We initially investigated miR-125b expression in the placenta tissues derived from PE patients using Real-time qPCR. Our results revealed that miR-125b levels was significantly elevated in both basal (Fig 1A) and chorionic (Fig 1B) plates of preeclamptic placentas compared to control placentas. We then further tested miR-125b levels in plasma derived from PE patients and the controls. In accordance with the placenta local expressions (Fig 1C), relative expression of circulating miR-125b was also significantly elevated in PE plasma. These results suggest that both local and circulating expression of miR-125b were significantly elevated in PE patients.

### SGPL1 is a direct target of PE-deregulated miR-125b

**Fig 2:**
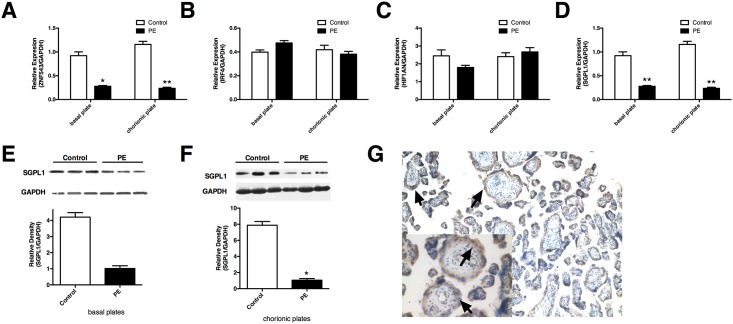
Predicted miR-125b downstream target genes in PE placentas. The mRNA levels of ZNF543 **(A)**, IRF4 **(B)**, SGPL1 **(C)** and HIF1AN **(D)** was detected using Real-time qPCR in placentas derived from PE patients and the controls. Protein expression of SGPL1 is reduced in both basal **(E)** and chorionic plates **(F)** preeclamptic placentas. **(G)** Immunohistochemistry assay was performed in paraffin sections of human placenta to show the localization of sphingosine-1-phosphate lyase 1 (SGPL1), arrows indicate trophoblast cells. Results are presented as mean ± SEM. Statistical comparison between miR-125b mimics (or inhibitor) and the corresponding NC was performed using SPSS, with p<0.05 considered as significant. *p < 0.05, **p < 0.01.

Identification of target genes is an essential step to study the association between miRNA and disease. We employed miRNA target prediction programs to search for miR-125b target genes, including miRanda, miRBase, TargetScan, miRDB and Pictar, and more than 1000 candidate downstream target genes of miR-125b were identified, such as ZNF543, HIF1AN, IRF4 and SGPL1 (Fig 2A–2D). We performed Real-time qPCR in placentas derived from PE patients and normal control pregnancies. Among these candidate target genes, we found that both the mRNA and protein levels of sphingosine-1-phosphate lyase 1 (SGPL1) were reduced in the basal and chorionic plates of preeclamptic placentas (Fig 2D, 2E and 2F) and showed an inverse correlation with miR-125b expression. SGPL1 is a key regulator for normal lipid metabolism, and altered SGPL1 expression results in severe developmental and functional defects[25]. We then conducted immunohistochemistry for SGPL1 in paraffin-embedded placental tissue sections, and observed that the positive signals mainly localized in the outer space of villous, most of which are trophoblast cells (Fig 2G).

**Fig 3:**
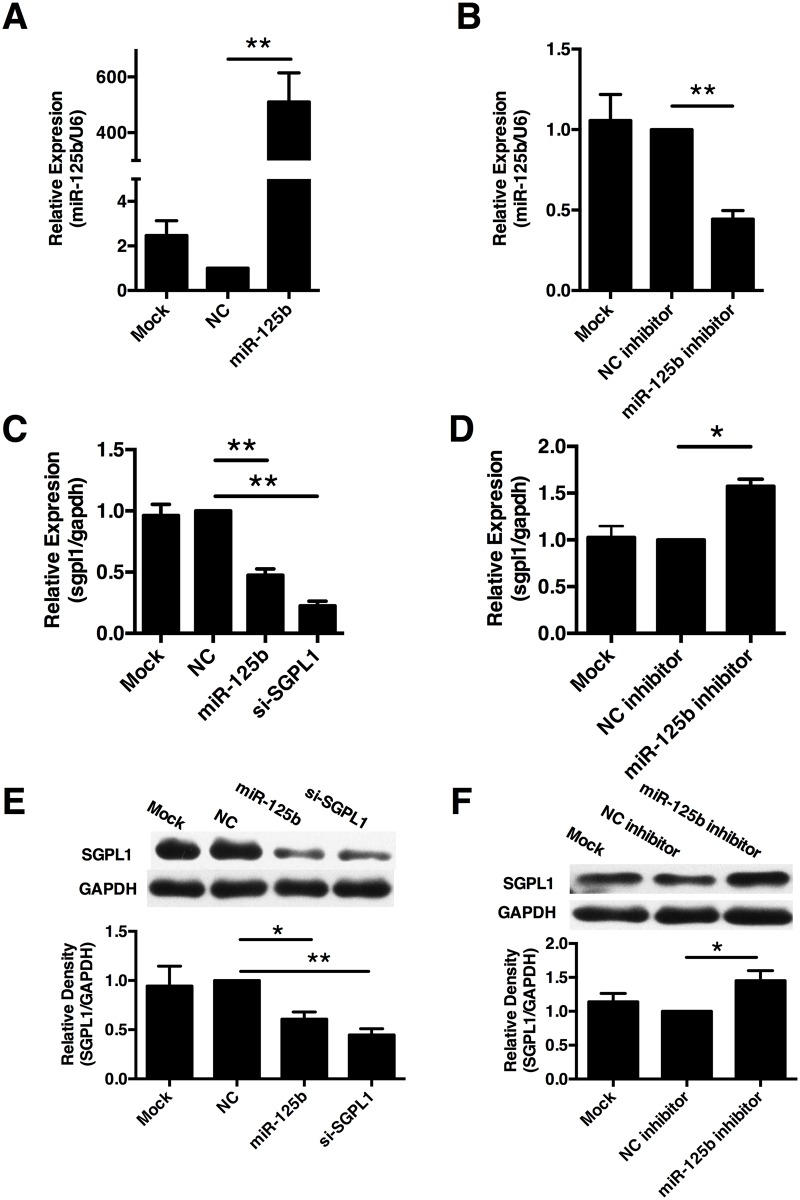
miR-125b negatively regulated SGPL1 in trophoblast cells. **(A)** The expression of miR-125b in HTR8/SVneo cells transfected with miR-125b mimics (miR-125b), scramble control (NC) or mock was revealed by Real-time qPCR. **(B)** The expression of miR-125b in HTR8/SVneo cells transfected with miR-125b inhibitor, scramble inhibitor (NC inhibitor) or mock was revealed by Real-time qPCR. Relative expression of miR-125b was normalized by U6. Both mRNA **(C)** and protein **(E)** levels of SGPL1 in HTR8/SVneo cells transfected with scramble control (NC) and miR-125b mimics (miR-125b) were revealed by Real-time qPCR and western blot. Both mRNA **(D)** and protein **(F)** levels of SGPL1 in HTR8/SVneo cells transfected with miR-125b inhibitor (miR-125b inhibitor) and scramble control inhibitor (NC inhibitor) were revealed by Real-time qPCR and Western blot. All experiments were repeated ≥3 times independently in identical conditions. Results are presented as mean ± SEM. Statistical comparison in separate groups between miR-210 and NC was performed using SPSS, with *p*<0.05 considered as significant. *p < 0.05, **p < 0.01.

We next focused on the regulation of SGPL1 by miR-125b in trophoblast cells. The human trophoblast cell line, HTR8/SVneo cells, was transfected with miR-125b mimics or miR-125b inhibitor and their corresponding scramble control separately. As present in Fig 2A and 2B, transfection of miR-125b mimics could significantly increase miR-125b levels in HTR8/SVneo cells, whereas miR-125b inhibitor could significantly reduce the endogenous miR-125b levels. We found that both mRNA and protein levels of SGPL1 were significantly reduced in HTR8/SVneo cells transfected with miR-125b mimics, as shown in Fig 3C and 3E, whereas inhibition of miR-125b reversed the suppressing effect on the expression of SGPL1 (Fig 3D and 3F). These results suggest that miR-125b negative-regulates SGPL1 expression in trophoblast cells.

**Fig 4:**
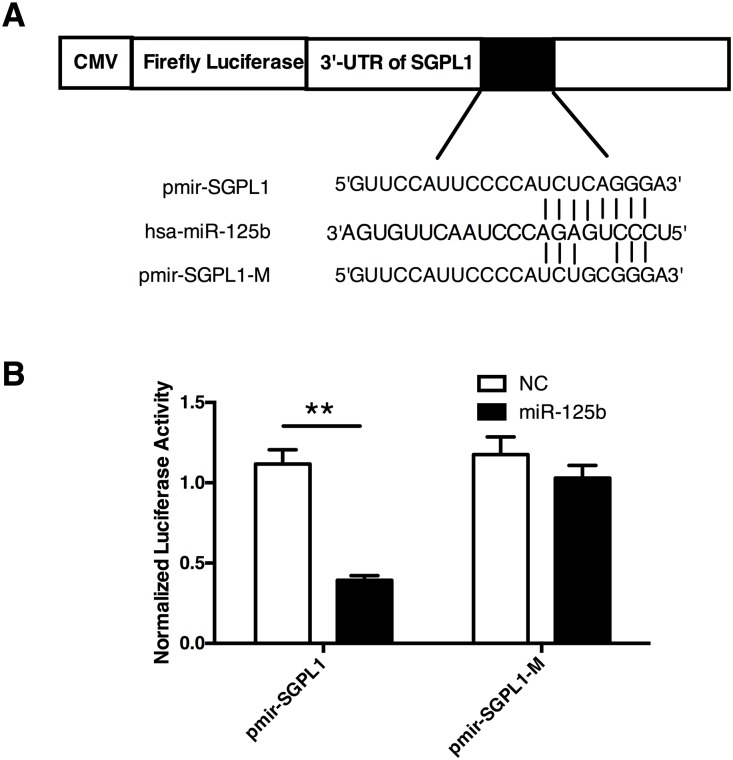
miR-125b directly targets 3’-UTR of SGPL1 transcript. **(A)** Schematic map of luciferase reporter assay constructs. The miR-125b target site within the 3’-UTR of SGPL1 was shown as black box. Sequences below indicated predicted miR-125b target site on wild-type (pmir-SGPL1) 3’-UTR, its mutated derivative (pmir-SGPL1-M), and the pairing region of miR-125b. **(B)** Luciferase assay in HTR8/SVneo cells transfected with pmir-SGPL1 and pmir-SGPL1-M reporter together with miR-125b or NC separately. The cells were harvested 48h later for luciferase assays. All experiments were repeated 3 times independently in identical conditions. Results are presented as mean ± SEM. Statistical comparison in separate groups between miR-210 and NC was performed using SPSS, with *p*<0.05 considered as significant. *p < 0.05, **p < 0.01.

The sequence of the 3’-UTR in SGPL1 mRNA contains a potential miR-125b binding site(Fig 4A). To determine whether SGPL1 is a direct target of miR125b, we generated two luciferase reporter constructs, one containing wild type 3’-UTR sequence of human SGPL1 (pmir-SGPL1) and the other containing mutant 3’-UTR of human SGPL1 to produce mutant luciferase reporter vector (pmir-SGPL1-M) (Fig 4A). The vectors were co-transfected into HTR8/SVneo cells with miR-125b mimics or the scramble control separately. As shown in Fig 4B, co-transfection of pmir-SGPL1 and miR-125b mimics significantly reduced the relative luciferase activity, whereas reduction of relative luciferase activity was not detected in cells expressing the mutant type vector pmir-SGPL1-M(Fig 4B). Over all, our results provide convincing evidence that miR-125b directly targets 3’-UTR of SGPL1 transcripts.

### miR-125b promotes interleukin-8 (IL-8) secretion in human trophoblast cells

**Fig 5:**
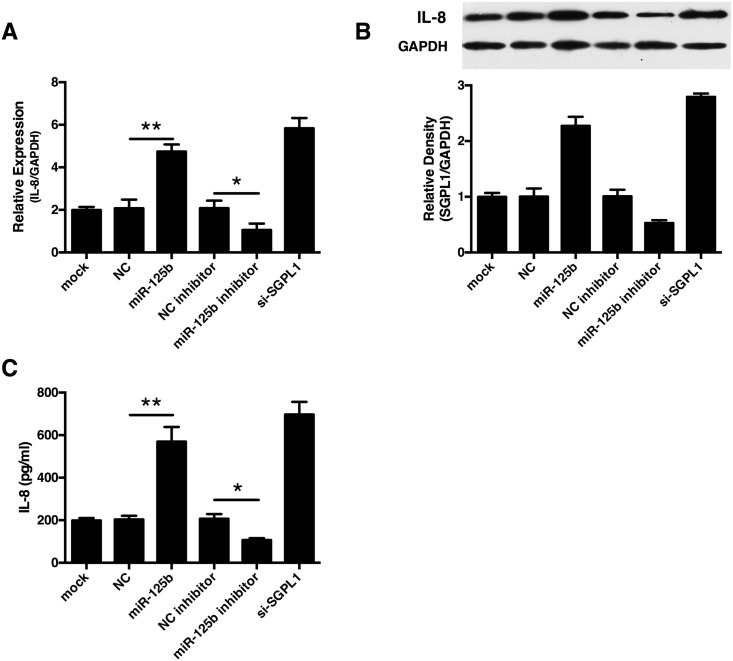
miR-125b enhanced IL-8 production in HTR8/SVneo cells. The human trophoblast cells, HTR/SVneo, were planted in 24-well plates (5×10^5^ cells/ml per well) (Corning, Tokyo, Japan). The culture supernatants were collected, filtered, and stored at –80°C until titration of IL-8. The expression of IL-8 in HTR8/SVneo cells transfected with miR-125b mimics, miR-125b inhibitor and SGPL1 siRNA (si-SGPL1) and their corresponding controls were tested using Real-time qPCR **(A)** and Western **(B)**. The concentrations of IL-8 in the supernatant of HTR8/SVneo cells were detected using ELISA **(C)**. All experiments were repeated 3 times independently in identical conditions. Results are presented as mean ± SEM. Statistical comparison in separate groups between miR-210 and NC was performed using SPSS, with *p*<0.05 considered as significant. *p < 0.05, **p < 0.01, ***p < 0.001.

One of the main symptoms observed early in pre-eclampsia is an imbalance of the immune system, where the impaired production of cytokines was observed. To further examine whether miR-125b and SGPL1 modulates cytokine productions, we took Real-time qPCR to screen the expression profile of cytokines. Our finally observed that transfection of miR-125b mimics or SGPL1 knockdown significantly increased the production of IL-8 in HTR8/SVneo cells compared with the scramble controls. In contrast, transfection of miR-125b inhibitor reduced production of IL-8 production(Fig 5A and 5B). We further conducted ELISA to determine IL-8 concentration in cell culture supernatant and found that IL-8 concentration was significantly elevated in cells transfected with miR-125b or siRNA for SGPL1(Fig 5C). Taken these results together, miR-125b significantly enhanced the production of IL-8 in trophoblast cells.

**Fig 6:**
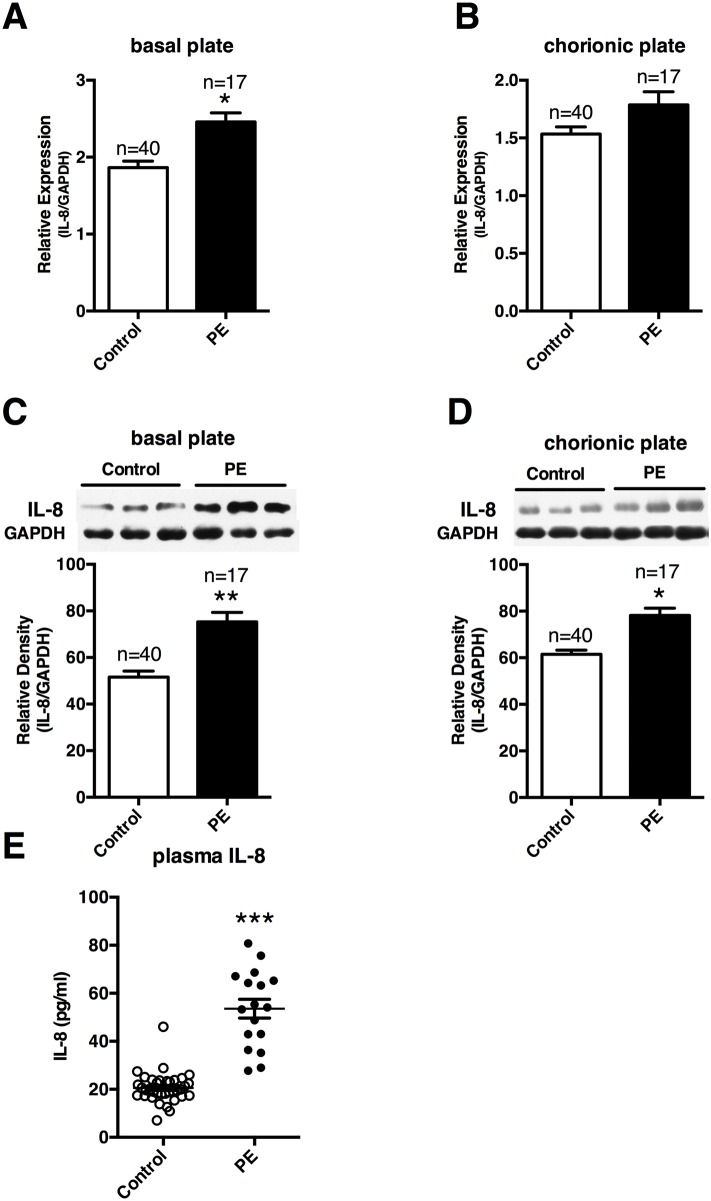
Circulating and placenta local expression of IL-8 in preeclampsia. mRNA levels and protein productions of IL-8 were tested using Real-time qPCR and Western respectively in the basal **(A, C)** and chorionic plates **(B, D)** of placenta tissues from women enrolled in our study. Circulating IL-8 in preeclampsia patients enrolled in our study was also examined using ELISA **(E)**. Results are presented as mean ± SEM. Statistical comparison between PE group and NC group was performed using SPSS, with p<0.05 considered as significant. *p < 0.05.

We next studied the expression of IL-8 in PE patients. We found that mRNA expression of IL-8 was increased significantly in basal, but not chorionic plates of preeclamptic placenta (Fig 6A and 6B), whereas production of IL-8 was significantly increased in both basal and chorionic plates of preeclamptic placentas (Fig 6C and 6D). We also tested IL-8 concentrations in the plasma, consist with previously reports[26], circulating IL-8 were also significantly elevated in PE patients enrolled in our study (Fig 6E). These results confirmed that both local and systemic levels of IL-8 were significantly elevated in PE patients.

### Overexpression of SGPL1 reverse the IL-8 secretion enhancing effect of miR-125b in HTR8/SVneo cells

**Fig 7:**
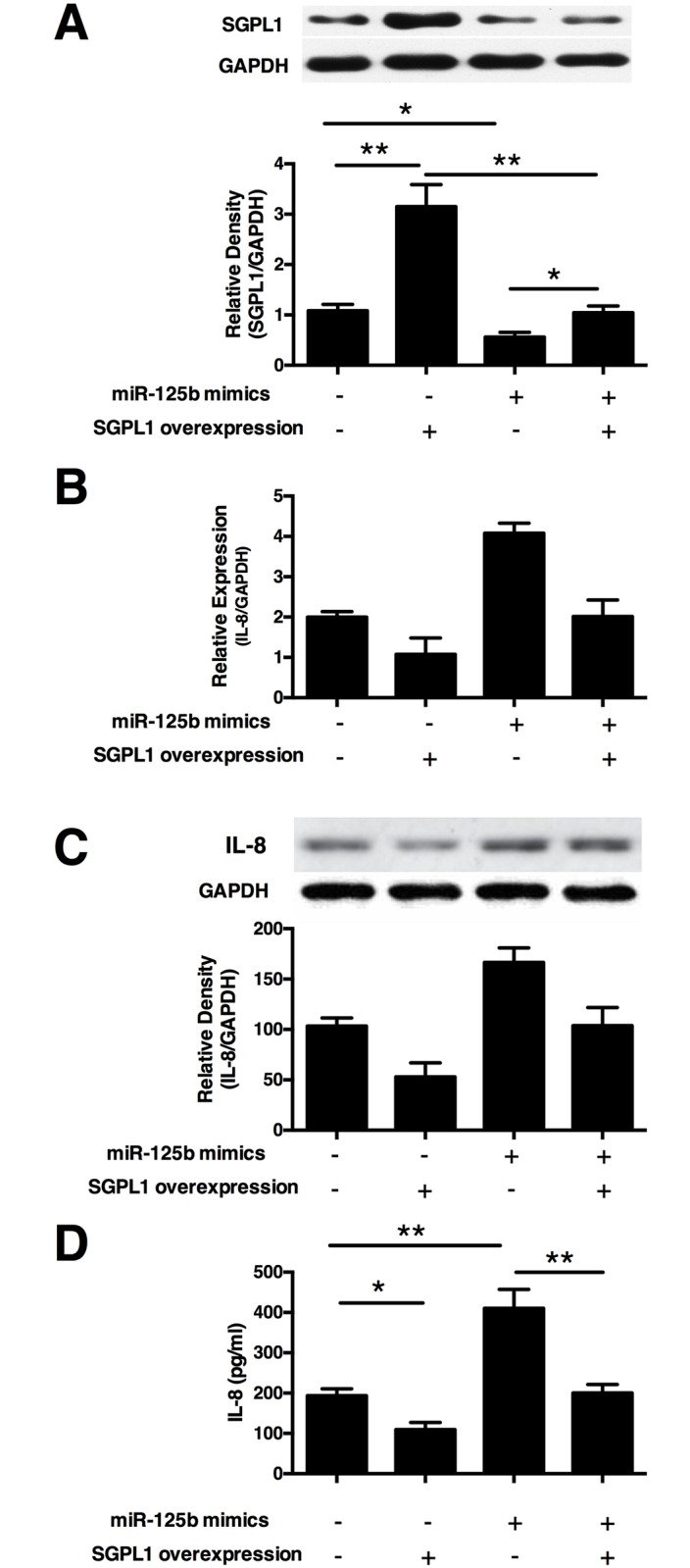
Sphingosine-1-phosphate receptor 1 (SGPL1) reversed the effect of miR-125b on the IL-8 production in HTR8/SVneo cells. **(A**) Relative expressions of SGPL1 were examined in HTR8/SVneo cells transfected with miR-125b and SGPL1 overexpressing pcDNA 4 vector (pSGPL1) alone or in combination, with scramble control (NC) or pcDNA 4 vector (pDNA4) as corresponding controls. The trophoblast cells, HTR8/SVneo, were transfected with miR-125b and SGPL1 overexpressing pcDNA 4 vector (pSGPL1) alone or in combination, with scramble control (NC) or pcDNA 4 vector (pDNA4) as corresponding controls. The mRNA **(B)**, protein **(C)** and secretion **(D)** of IL-8 were tested using Real-time PCR, Western and ELISA separately. All experiments were repeated ≥3 times independently in identical conditions. Results are presented as mean ± SEM, with *p*<0.05 considered as significant. *p < 0.05, **p < 0.01.

To clarify whether SGPL1 is directly involved in IL-8 secretion-enhancing effect of miR-125b, we transfected HTR8/SVneo cells with miR-125b mimics together with an SGPL1 overexpressing vector (pSGPL1) to carry out a rescue experiment, as shown in Fig 7A. The mRNA and protein expression of IL-8 was determined by Real-time qPCR and Western Blot. We found that miR-125b could significantly increase IL-8 expression, whereas overexpression of SGPL1 reversed the enhancing effect, as shown in Fig 7B and 7C. We further tested the concentration of IL-8 in the cell culture supernatant and observed a similar effect as previously (Fig 7D). Taken together these results provide evidence that SGPL1 is involved in the IL-8 secretion-enhancing effect of miR-125b in human trophoblast cells.

## Discussion

PE is pregnancy-specific syndrome and one of the leading cause of maternal and fetal mortality and morbidity worldwide[27]. The exact etiology of PE is still unclear, despite intense investigations. It has been reported that an altered trophoblast invasion, aberrant angiogenic factors, enhanced inflammatory response, and oxidative stress, in maternal circulation, are frequently associated with development of this disorder[28–31]. However, the molecular mechanism underlying these events remains poor understood.

miRNAs have been indicated to play essential roles in the development and disease[32]. Several reports have suggested that expression profiles of miRNAs in the circulation and placenta of PE patients are deregulated[10–13,33]. Recent studies revealed that many of them were involved in the regulation of angiogenesis, inflammation and trophoblast cell migration/invasion[34–40]. Thus far, it remains to be clarified what roles the dysregulated miRNAs play in the pathology of PE.

miR-125 family had two homologues miR-125a and miR-125b in human, MiR-125a has been found to be transcribe from the loci located at 19q13, while miR-125b is verified to be transcribed from two loci located on chromosomes 11q23(miR-125b-1) and 21q21(miR-125b-2). mature miR-125b (short for miR-125b-5p) arises from the 5'-arm of miR-125b-1 and miR-125b-2[41].

In this study, we observed aberrant miR-125b expression in preeclamptic placentas. miR-125b was significantly upregulated in both basal and chorionic plates of preeclamptic placentas than in that of the control placentas. miR-125b was reported to play an essential role for the immune response or hypoxia[42]. It is implicated to be involved in proliferation, apoptosis, invasion and VEGF production and is considered to be potential biomarkers and therapeutic targets for different diseases[41]. However, it is unclear whether miR-125b has a role in human placenta.

miRNAs usually play their critical roles by repressing the expression of target gene transcripts[43]. miR-125b has been reported to target many genes including c-Jun, ENPEP, CK2-α, p53, CCNJ, and MEGF9[17,44–46]. Several clues in this study prove that SGPL1 is a novel direct target of miR-125b in trophoblast cells. First, SGPL1 expression is negatively correlated with miR-125b in the placenta, and SGPL1 localized in trophoblast cells similar as miR-125b[20,21]. Second, SGPL1 expression could be reduced by transfection of miR-125b mimics in HTR8/SVneo cells, and luciferase assay suggested a direct interaction between miR-125b and SGPL1. Third, overexpression of SGPL1 could reverse the IL-8 production enhancing effect of miR-125b in trophoblast cells. Taken together, these results provide strong evidence that SGPL1 is a direct target of miR-125b, and participate in mediating the IL-8 secretion enhancing effect of miR-125b in trophoblast cells.

SGPL1 catalyzes the lysis of S1P, which functions in various biological processes such as inflammation, neovascularization and cell growth and survival through its G protein–coupled receptors (S1P1-5)[47]. Suppression of SGPL1 gene impaired the degradation of S1P, leading to significantly increased S1P levels, and influent the sphingolipid metabolic pathway on lipid homeostasis. Recent reports show that SGPL1 deficiency results in elevated levels of pro-inflammatory cytokines in mice by impairing S1P/S1PRs axis[48].

Placenta produces a large amount of pro- and anti-inflammatory cytokines and cytokine-like angiogenic growth factors. Dysregulated placental production of immunomodulators was considered to be important aspects in the aetiology of the syndrome[31]. Therefore, based on previous SGPL1 deficiency studies[48], we tested the effect of miR-125b on the cytokine secretion of trophoblast cells. Our results demonstrated that transfection of miR-125b mimics enhances IL-8 secretion of HTR8/SVneo cells, while transfection of miR-125b inhibitor inhibits IL-8 secretion. These finding indicate that miR-125b could promotes IL-8 production of trophoblast cells.

Based on the above studies, we further investigated circulating and local IL-8 levels in clinical samples. We found that both circulating and local IL-8 levels were increased in PE patients than that in the controls. Interestingly, these observations were in accordance with our *in vitro* results, and also in line with previous reports[49,50].

IL-8 (Interleukin-8) is one of the main chemokines for neutrophils and T lymphocytes that produced by a number of tissues and cells; it has been demonstrated to be involved in the regulation of pathological angiogenesis, endothelial activation and cell migration/invasion[51]. Increased production of IL-8 provides a concentration gradient to recruit more neutrophils and T lymphocytes[52]. These cells can adhere on the endothelial cells, invade into sites of inflammation and release a variety of enzymes and cytokines, leading to inflammation. From this perspective, the increased IL-8 production in trophoblast cells may play a role in the pathogenesis of PE by recruitment of excess neutrophils to increase local inflammation.

In current study, we excluded all women with any other medical illnesses, such as gestational diabetes and preexisting hypertension, which have been reported to increase the risk for PE, thus making the validation of miRNAs more specific. As far as we know, it is the first time that miR-125b was validated to target SGPL1 to promote IL-8 production in PE. However, some questions remain to be answered. First, as this was a case-control study, a causal relationship between miR-125b and risk of PE could not be confirmed to exist. Second, besides trophoblast cells, placenta is also comprised of decidual cells, endothelial cells, immune cells and many other cells. Further investigation should be performed to clarify if miR-125b plays a role in the regulation of other cells in the placenta.

In summary, our study provides new evidence that elevated miR-125b significantly enhances IL-8 production in the placenta via decreasing SGPL1 expression. This pathway contributes to the high levels of IL-8 in the circulation and placenta of PE patients. Therefore, miR-125b and SGPL1 may be developed to be potential clinical predictive and therapeutic targets for PE.
